# Transcriptomic evidence for distinct mechanisms underlying abscission deficiency in the *Arabidopsis* mutants *haesa/haesa*-*like 2* and *nevershed*

**DOI:** 10.1186/s13104-018-3864-x

**Published:** 2018-10-23

**Authors:** Isaiah Taylor, John C. Walker

**Affiliations:** 1 Division of Biological Sciences, University of Missouri, Columbia, MO 65211 USA; 2 Interdisciplinary Plant Group, University of Missouri, Columbia, MO 65211 USA; 3 Department of Statistics, University of Missouri, Columbia, MO 65211 USA; 4 Present Address: Department of Biology and Howard Hughes Medical Institute, Duke University, Durham, NC 27708 USA

**Keywords:** Abscission, HAESA, HAESA-LIKE2, NEVERSHED

## Abstract

**Objective:**

In *Arabidopsis*, the abscission of floral organs is regulated by two related receptor-like protein kinases, HAESA and HAESA–like 2 (HAE/HSL2). Signaling by HAE/HSL2 leads to expression of genes encoding secreted cell wall remodeling and hydrolase enzymes. *hae hsl2* mutants fail to induce expression of these genes and retain floral organs indefinitely. Mutants in the gene *NEVERSHED (NEV)* also fail to abscise floral organs and phenotypically resemble *hae hsl2*. *NEV* encodes an ADP-ribosylation factor GTPase-activating protein that localizes to the trans-Golgi network and early endosome. *nev* displays altered Golgi morphology and aberrations in vesicular trafficking. The mechanism by which *nev* fails to abscise is presently unknown. It has been hypothesized that *nev* fails to activate HAE/HSL2 signaling. In this study we use RNA-Sequencing to test this hypothesis.

**Results:**

We show that the transcriptional alterations in *hae hsl2* and *nev* are highly divergent. *hae hsl2* displays a clear reduction in expression of genes associated with cell wall remodeling and pectin degradation, while *nev* displays vast transcriptional changes associated with response to pathogens. These results suggest that the mechanism of the defect between *hae hsl2* and *nev* are distinct.

**Electronic supplementary material:**

The online version of this article (10.1186/s13104-018-3864-x) contains supplementary material, which is available to authorized users.

## Introduction

In plants, abscission is the programmed shedding of entire organs resulting from environmental stimuli or an endogenous developmental program. Post-pollination abscission of sepals, petals, and stamen in *Arabidopsis* has emerged as an important model developmental signaling system. Floral abscission is regulated by the redundant receptor protein kinases HAESA and HAESA-LIKE 2 [1, 2]. Double *hae/hsl2* mutants fail to activate an intracellular signaling pathway controlling abscission and their floral organs fail to abscise [1, 3].

In addition to plants defective in the *HAE/HSL2* signaling pathway, mutants in the ADP-ribosylation factor GTPase-activating Protein (Arf-GAP) encoding *NEVERSHED* display a strong abscission defect [4]. Analysis of floral abscission zone cells shows that these *nev* mutants display aberrant Golgi morphology and over-accumulation of paramural vesicles, suggesting disruption of the secretory system [4]. The exact molecular defect causing abscission deficiency in the *nev* mutant is not known. It has been hypothesized that *nev* may fail to activate intracellular signaling mediated by HAE/HSL2 [5]. In this study, we utilized RNA-sequencing to compare the transcriptome of a *hae hsl2* mutant and a *nev* mutant in order to test this hypothesis.

## Main text

### Materials and methods

#### Lines used in this study

The *nev*-*3* mutant, isolated in Ler and outcrossed to Col-0, and the *nev*-*3 serk1*-*5* suppressor in the outcrossed *nev*-*3* background were kindly provided by Dr. Sarah Liljegren [4, 6]. *hae*-*3 hsl2*-*3* has been previously described, and is deposited at ABRC.

#### Plant growth conditions

Plant were grown in a 16 h light cycle, ~ 700 lx, at 22 °C, ~ 65% relative humidity, and fertilized once at 3 weeks post-germination with 1 × strength Miracle-Gro (Scotts Miracle-Gro Company).

#### RNA-sequencing analysis

A minimum of 8 and maximum of 15 mid-stage 15 floral receptacles were isolated and pooled per biological replicate. Three biological replicates per genotype were prepared in each experiment. RNA was isolated using the Trizol reagent (Life Technologies). Each receptacle was dissected by taking a 1 mm section of floral tissue comprised of 1/3 mm stem and 2/3 mm receptacle. Libraries were created using the TruSeq mRNA Library Prep Kit (Illumina). Libraries from each experiment were individually barcoded, pooled, and run on a single lane of Illumina Sequencing using the NextSeq 500 instrument.

Reads were mapped to The Arabidopsis Information Resource (TAIR) 10 gene sequences and quantified using TopHat (v. 2.9) and Cufflinks (v2.1.1) [7]. We utilized default settings for alignments, and performed differential expression analyses using cuffdiff with default settings. Data were analyzed and visualized in R using CummeRbund and ggplot2 [7]. GO analysis was performed utilizing AgriGo [8]. Differential expression was chosen to denote an FDR < .05 from cufflinks output.

### Results

#### RNA-sequencing of abscission deficient mutants *hae*-*3 hsl2*-*3* and *nev*-*3*

**Fig. 1:**
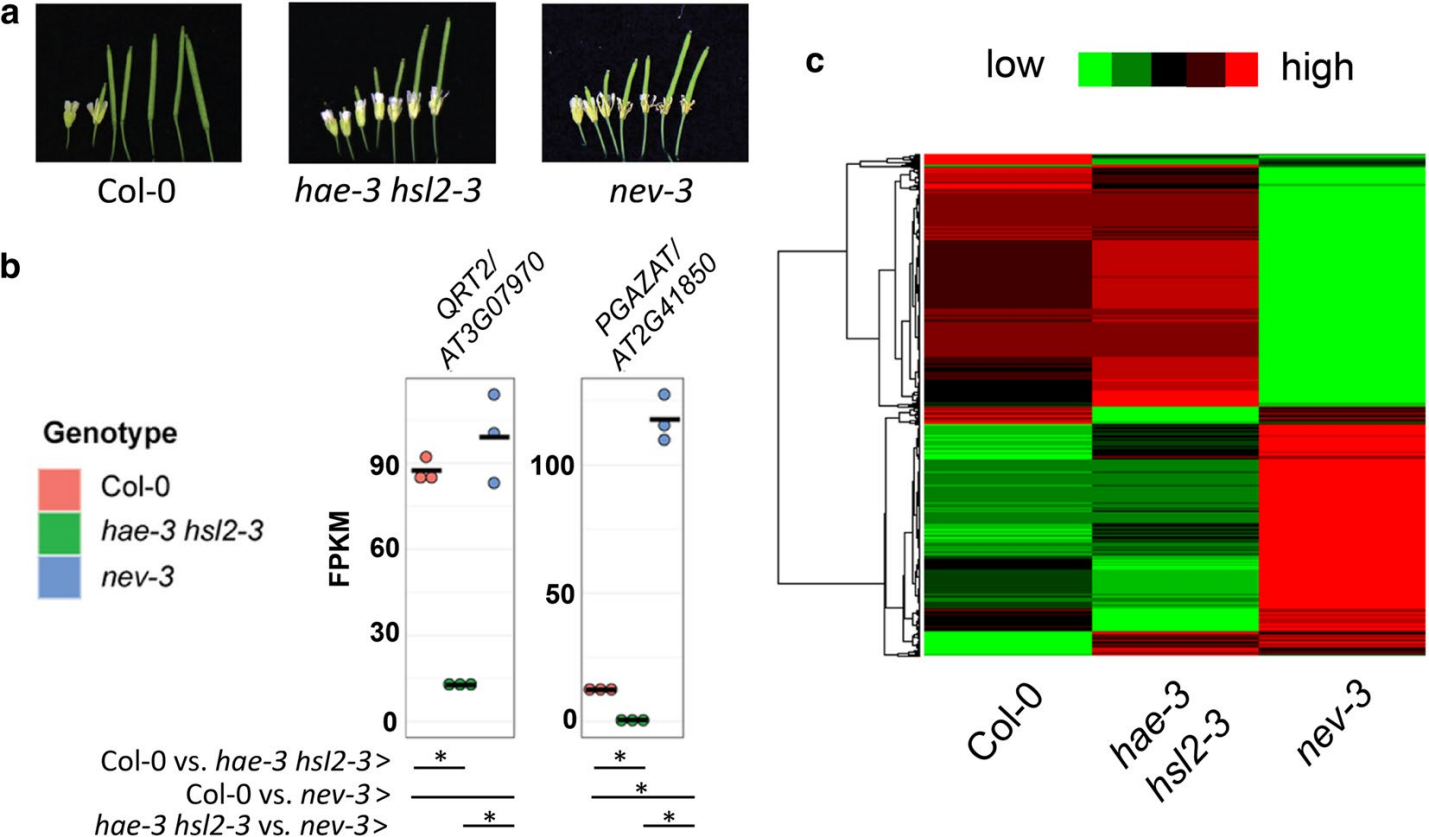
RNA-Sequencing of *hae hsl2* and *nev.* **a** Abscission phenotype of wildtype Col-0, *hae*-*3 hsl2*-*3,* and *nev*-*3.* **b** Transcript abundance measurements for abscission associated polygalacturonase genes. Points represent FPKM values per individual replicates of the indicated genotype. Asterisks below represent statistical significance at FDR < .05. **c** Heatmap of differentially expressed genes between Col-0, *hae*-*3 hsl2*-*3,* and *nev*-*3*

*nev* mutants are abscission deficient and resemble *hae hsl2* where floral organs remain strongly attached to the developing fruit [4] (Fig. 1a). To investigate the potential mechanistic relationship between these two mutants, we performed RNA-Sequencing comparing wildtype Col-0 and the two previously described abscission deficient mutants *hae*-*3 hsl2*-*3* and *nev*-*3* [4, 9]. In this study, we sampled RNA derived from mid-stage 15 floral receptacles. Stage 15 is the floral developmental stage where pollination has occurred and abscission signaling has been initiated, but floral abscission has not been completed [3] (Additional file 1: Figure S1).

After RNA-Sequencing, we assessed transcript abundance measurements for genes known to be regulated by *HAE/HSL2* [output of differential expression analysis for RNA-Sequencing experiments included in Additional file 2: Dataset 1. Previous studies have identified two genes encoding secreted polygalacturonases *QRT2* and *PGAZAT* with a partially abscission defective double mutant phenotype and whose expression is regulated by *HAE/HSL2* [3, 10]. These genes are hypothesized to contribute to the loosening of the cell wall at the site of abscission [11]. While these genes are a non-exhaustive sample of those likely regulated by HAE/HSL2, they serve as a reliable indicator or pathway activity. Their transcript abundance measurements are plotted in Fig. 1b.

The results confirm a significant reduction in *hae*-*3 hsl2*-*3* in comparison to Col-0 for both *QRT2* and *PGAZAT* (Fig. 1b). In contrast, *nev*-*3* showed a transcript abundance level statistically similar to wildtype for *QRT2*, but a dramatic > 8 fold increase in *PGAZAT* transcript abundance. These data suggest that, unlike *hae*-*3 hsl2*-*3, nev*-*3* does not exhibit generally reduced abscission signaling, but surprisingly exhibits strong and apparently dysregulated expression of key *HAE/HSL2* regulated genes.

Global differential expression and gene ontology (GO) analysis demonstrated statistically significant differences of 1055 and 326 genes in the Col-high/*hae*-*3 hsl2*-*3*-low and Col-low/*hae*-*3 hsl2*-*3*-high comparisons, respectively. GO analysis identified moderate enrichment in terms such as r*esponse to stimulus, response to stress, cell wall*, and other terms similar to those previously associated with gene expression changes in the *hae hsl2* mutant [full results for all GO analyses included in Additional file 3: Dataset 2. In contrast, *nev*-*3* shows evidence of dramatic transcriptional reprogramming, with 4627 and 4753 statistically significantly different genes in the Col-high/*nev*-*3*-low and Col-low/*nev*-*3*-high comparisons, and 4861 and 5079 statistically significantly different genes in the *hae*-*3 hsl2*-*3*-high/*nev*-*3*-low and *hae*-*3 hsl2*-*3*-low/*nev*-*3*-high comparisons. A heat map displaying relative levels of all differentially expressed genes between the three genotypes is displayed in Fig. 1c. GO analysis of genes with higher expression in *nev* identified extremely significant enrichment of terms such *response to stress, response to biotic stimulus*, *kinase activity, cell death,* and other terms associated with response to biotic stress. There is less of a clear pattern to the terms enriched in genes in the Col-high/*nev*-*3* low comparison. Potentially informative terms include *lipid metabolic process* and *endomembrane system*.

Overall, these results are surprising in that the pattern of abscission associated polygalacturonase gene expression in *nev*-*3* bears little resemblance to that of *hae*-*3 hsl2*-*3*. There also appears to be general transcriptional reprogramming in the *nev* mutant and over-activation of pathogen response signaling. These results suggest the mechanism of abscission defect in *nev* is dissimilar to that of *hae hsl2*.

#### RNA-sequencing of *nev*-*3* and *nev*-*3 serk1*-*5*

**Fig. 2:**
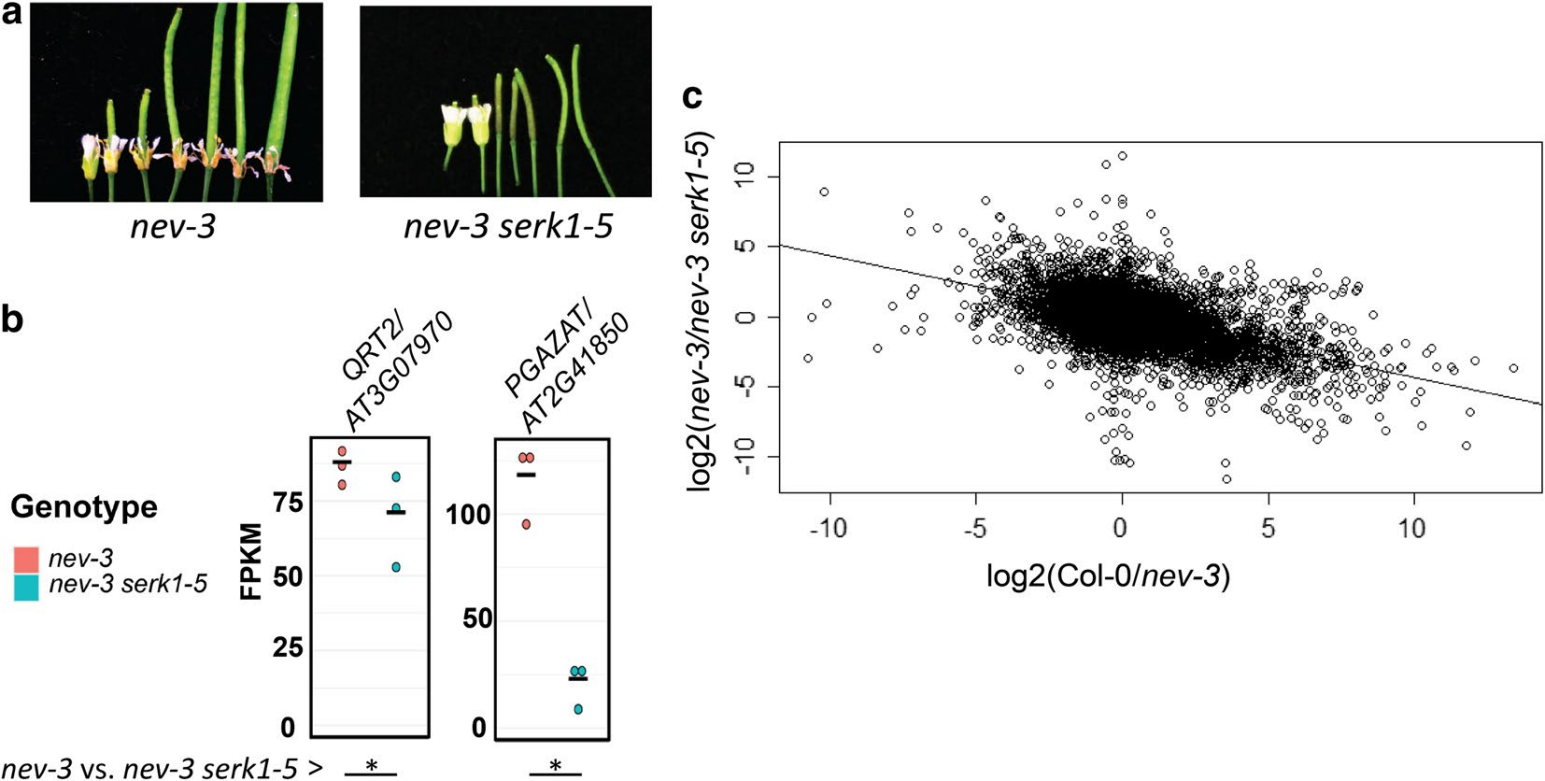
RNA-Sequencing of *nev* and *nev serk1.* **a** Abscission phenotype of *nev*-*3* and *nev*-*3 serk1*-*5.* **b** Transcript abundance measurements for abscission associated polygalacturonase genes. Points represent FPKM values per individual replicates of the indicated genotype. Asterisks below represent statistical significance at FDR < .05 for the indicated comparisons. **c** Plot of linear regression modelling log2(fold change) of Col-0/*nev*-*3* measurement against *nev*-*3/nev*-*3 serk1*-*5* gene expression measurements

Results of a *nev* suppressor screen have shown that loss of function mutations of *SERK1* are capable of suppressing the abscission defect and subcellular Golgi defects of *nev* [6] (Fig. 2a). *SERK1,* along with the related *SERK2, SERK3/BAK1*, and *SERK4*, have been shown to positively regulated abscission by encoding HAE/HSL2 co-receptors [12, 13]. It has not been clear how mutating a positive regulator of abscission signaling can suppress the *nev* abscission defect.

Considering the results of the *nev* RNA-Seq experiment above, we hypothesized there may be a *SERK1* regulated signaling pathway over-activated in *nev* that leads to transcriptional reprogramming and that interferes with abscission by an unknown mechanism. We further hypothesized that mutating *SERK1* may lead to suppression of the abscission defect of *nev* by reducing signal strength of this pathway. To test this hypothesis, we performed RNA-Sequencing to compare *nev*-*3* to the previously described double loss of function suppressor mutant *nev*-*3 serk1*-*5* [6].

We first examined expression of *QRT2* and *PGAZAT. nev*-*3 serk1*-*5* displays a slightly lower level of transcript abundance for *QRT2*, and a > 5 fold reduction in transcript abundance of *PGAZAT* (Fig. 2b). These data are consistent with a model whereby mutation in *SERK1* suppresses the dysregulated expression in *nev*-*3.*

We further performed global gene expression analysis and GO term enrichment in a similar manner as above. We observed 4756 genes with a statistically significantly higher transcript abundance in *nev*-*3* compared to *nev*-*3 serk1*-*5,* and 4848 genes with a lower transcript abundance in *nev*-*3* compared to *nev*-*3 serk1*-*5*. GO term analysis of genes with higher levels in *nev*-*3* compared to *nev*-*3 serk1*-*5* shows that the top terms in the Biological Process category include *response to stimulus, response to stress, multi*-*organism process, cell death,* and other terms associated with biotic stress response (Additional file 2: Dataset 2). 8 of the top 10 terms in this comparison are identical to those in the top 10 Biological Process terms associated with genes higher in *nev*-*3* compared to Col-0. These data suggest that in *nev*-*3 serk1*-*5*, there is a reduction in an intracellular signaling pathway and are consistent with a model where the defect of *nev* is caused by over-activation of *SERK1* signaling.

Finally, to test the hypothesis that mutation of *SERK1* can reverse gene expression changes in *nev,* we performed linear regression on the log(fold change) values from the Col-0/nev-3 comparison versus those from the *nev*-*3/nev*-*3 serk1*-*5* comparison. We hypothesized that we would observe an inverse relationship between these log(fold change) measurements. Consistent with this expectation, we observe a highly statistically significant negative relationship, indicating that gene expression changes in *nev*-*3* compared to wildtype tend to be offset in *nev*-*3 serk1*-*5* (Fig. 2c). This result is further consistent with the hypothesis that mutation of *SERK1* reduces the signal strength of the over-active signaling process in *nev.*

### Conclusion

Overall, these data suggest that in *nev*-*3* there is over-activation of *SERK1* mediated signaling that leads to widespread transcriptional reprogramming and consequent cellular dysfunction in the abscission zone, leading to an abscission defect. Mutation of *SERK1* likely reduces the strength of this over-activated signal and allows abscission to occur. Determination of what effect this mis-regulated signaling has on abscission zone function is a direction for future research. One possibility was illuminated by a recent report demonstrating that floral abscission zone cells exhibit a regulated lignification pattern, and that the *nev* mutant exhibits disorganized and ectopic lignification [14]. It is possible this ectopic lignification underlies the phenotype of *nev,* perhaps by interfering with the normal cell separation processes that occur as a result of the modification of abscission zone cell walls and dissolution of the middle lamella. In this scenario, the *nev* phenotype and associated transcriptomic changes could occur by activation of *SERK1* signaling that aberrantly feeds into a lignification pathway, as well as pathways regulating abscission related hydrolases such as *PGAZAT* and pathogen response genes. This model presumes the inhibitory effect on abscission of ectopic lignification overrides the effect of strong abscission hydrolase expression. This model will require substantial additional detailed genetic and physiological investigation. However, its strength is that it offers an explanation for two counter-intuitive properties of the *nev* mutant: that *nev* exhibits high levels of expression of hydrolase genes such as *PGAZAT* and *QRT2,* and that the *nev* phenotype can be suppressed by mutation of *SERK1,* a positive regulator of abscission signaling [4, 6].

## Limitations

In this paper we show that the *nev* mutant exhibits widespread transcriptional reprogramming, including a stark increase in pathogen response gene expression, and that reducing this transcriptional response by mutating *SERK1* suppresses the abscission defect of *nev.* However, this work does not provide a fundamental physiological explanation for why *nev* does not abscise, nor does it point to a mechanism by which *SERK1* signaling is activated in the *nev* mutant. It only presents evidence at the transcriptional level that the mechanism of abscission deficiency between *hae hsl2* and *nev* appears to be quite distinct. Further work on the *nev* mutant and its suppressors will be required to more fully understand this phenomenon.

## Additional files

**Additional file 1: Figure S1.** Stage 15 flowers.

**Additional file 2: Dataset 1.** Output of differential expression analysis.

**Additional file 3: Dataset 2.** Output of GO analysis.

## Abbreviations

HAE: HAESA
HSL2: HAESA-LIKE 2
NEV: NEVERSHED
SERK1: somatic embryogenesis receptor-like kinase 1
PGAZAT: polygalacturonase from the abscission zone of *Arabidopsis thaliana*
QRT2: QUARTET2

## References

[1] Cho SK, Larue CT, Chevalier D, Wang H, Jinn T-L, Zhang S (2008). Regulation of floral organ abscission in *Arabidopsis thaliana*. Proc Natl Acad Sci USA..

[2] Stenvik G-E, Tandstad NM, Guo Y, Shi C-L, Kristiansen W, Holmgren A (2008). The EPIP peptide of INFLORESCENCE DEFICIENT IN ABSCISSION is sufficient to induce abscission in *Arabidopsis* through the receptor-like kinases HAESA and HAESA-LIKE2. Plant Cell..

[3] Niederhuth CE, Patharkar OR, Walker JC (2013). Transcriptional profiling of the *Arabidopsis* abscission mutant hae hsl2 by RNA-Seq. BMC Genomics..

[4] Liljegren SJ, Leslie ME, Darnielle L, Lewis MW, Taylor SM, Luo R (2009). Regulation of membrane trafficking and organ separation by the NEVERSHED ARF-GAP protein. Development..

[5] Liljegren SJ. Organ abscission: exit strategies require signals and moving traffic. Curr Opin Plant Biol. 2012. http://www.sciencedirect.com/science/article/pii/S1369526612001252. Accessed 15 Jan 2014. 10.1016/j.pbi.2012.09.012 23047135

[6] Lewis MW, Leslie ME, Fulcher EH, Darnielle L, Healy PN, Youn J-Y (2010). The SERK1 receptor-like kinase regulates organ separation in *Arabidopsis* flowers. Plant J Cell Mol Biol..

[7] Trapnell C, Roberts A, Goff L, Pertea G, Kim D, Kelley DR (2012). Differential gene and transcript expression analysis of RNA-seq experiments with TopHat and Cufflinks. Nat Protoc.

[8] Du Z, Zhou X, Ling Y, Zhang Z, Su Z (2010). agriGO: a GO analysis toolkit for the agricultural community. Nucleic Acids Res..

[9] Niederhuth CE, Cho SK, Seitz K, Walker JC (2013). Letting go is never easy: abscission and receptor-like protein kinases. J Integr Plant Biol.

[10] Ogawa M, Kay P, Wilson S, Swain SM (2009). ARABIDOPSIS DEHISCENCE ZONE POLYGALACTURONASE1 (ADPG1), ADPG2, and QUARTET2 are polygalacturonases required for cell separation during reproductive development in *Arabidopsis*. Plant Cell..

[11] Patharkar OR, Walker JC (2018). Advances in abscission signaling. J Exp Bot.

[12] Meng X, Zhou J, Tang J, Li B, de Oliveira MV, Chai J (2016). Ligand-induced receptor-like kinase complex regulates floral organ abscission in *Arabidopsis*. Cell Rep..

[13] Santiago J, Brandt B, Wildhagen M, Hohmann U, Hothorn LA, Butenko MA (2016). Mechanistic insight into a peptide hormone signaling complex mediating floral organ abscission. Elife..

[14] Lee Y, Yoon TH, Lee J, Jeon SY, Lee JH, Lee MK (2018). A lignin molecular brace controls precision processing of cell walls critical for surface integrity in *Arabidopsis*. Cell.

