# Supplementary figures and images for: Transcriptomic evidence for distinct mechanisms underlying abscission deficiency in the Arabidopsis mutants haesa/haesa-like 2 and nevershed
Source: BMC Res Notes. 2018 Oct 23;11:754. doi: 10.1186/s13104-018-3864-x (PMC6199728; doi:10.1186/s13104-018-3864-x)

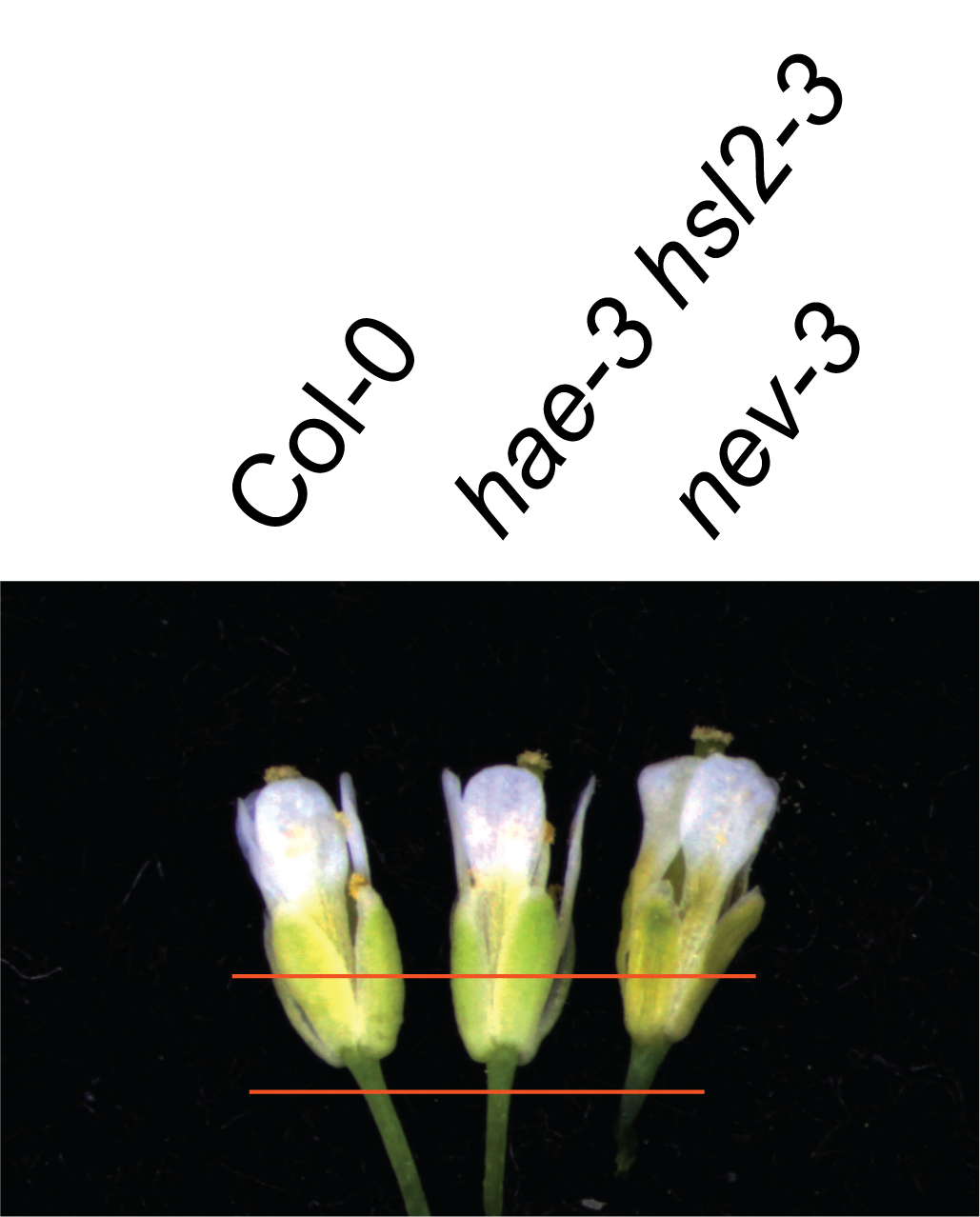

Supplement: Supplementary file 1 — Additional file 1: Figure S1. Stage 15 flowers. [file 13104_2018_3864_MOESM1_ESM.png]
